# The Impact of Inflammatory Bowel Disease on Pregnancy and the Fetus: A Literature Review

**DOI:** 10.7759/cureus.5648

**Published:** 2019-09-13

**Authors:** Hira Pervez, Norina Usman, Munis M Ahmed, Mydah S Hashmi

**Affiliations:** 1 Internal Medicine / Cardiology, Dow University of Health Sciences (DUHS), Karachi, PAK; 2 Internal Medicine, Veterans Affairs Palo Alto Health Care System - Stanford University School of Medicine, Palo Alto, USA; 3 Internal Medicine, St Mary Mercy Livonia Hospital, Livonia, USA; 4 Internal Medicine, Army Medical College, Rawalpindi, Islamabad, PAK

**Keywords:** inflammatory bowel disease, pregnancy, fetus, crohn’s disease, ulcerative colitis, complications, impact, ibd, chronic bowel disease, maternal complications

## Abstract

Inflammatory bowel disease (IBD) is a constellation of devastating chronic inflammatory changes in the bowel, either involving the large or small bowel or part of both. As it is widely diagnosed in the fertile age group, this disorder can present itself, very commonly, during pregnancy and thus a better understanding of the disease can be an important factor to influence the maternal and fetal well-being. Medications are what is considered the first line in the management of this disease to control the symptoms or keep the disease in remission. In addition to this, the drugs used to keep the disease in remission can also cause significant adverse effects on the patient and the new nurturing life preparing itself for the outside world. What the fetus gets from the mother will stay for life with the child. We conducted an electronic literature review search which highlights the significance and impact of sustained remission of IBD and the cautious use of various drugs during pregnancy for that purpose. In addition to the influences already mentioned, It is evident that nutritional deficiencies can also prevail with the advancing disease, something to manage as a side note as well. These deficiencies can have a definite effect on the fetus and may cause developmental malformations. In order to avoid this process, a systemic and joint approach should be curtailed. This can reduce the adverse outcomes associated with this ailment during pregnancy.

## Introduction and background

Inflammatory Bowel Disease (IBD) is a spectrum of chronic debilitating inflammatory conditions of a relapsing and remitting nature. The disease is devastating due to its symptoms including diarrhea/constipation, blood in stools, increased bowel movements, abdominal cramps, nausea, and vomiting. In addition to the gastrointestinal (GI) symptoms, fever, weight loss, arthralgia, and malaise are few other frequent systemic symptoms. Pregnancy remains a physiological and emotional period for a woman. Diseases that can lead to complications, both maternal and fetal, can cause an impact on the overall morbidity and mortality of the woman. In addition to this, the usage of drugs can also have a role in the adverse effects on the fetus as well as the mother. This article discusses in detail the impact of IBD on pregnancy and its outcomes.

A thorough electronic literature review search was conducted using PubMed and Google Scholar. The keywords incorporated were “maternal complications”, “fetal complications”, “inflammatory Bowel Disease”, and "IBD" as MeSH terms. The articles reviewed were preferably in English, peer-reviewed between January 2015-August 2019. In order, to provide supporting information, additional literature was obtained from the reference citations from the initial literature search. A detailed review of the titles and abstracts was done to exclude any information outside the scope of our review. The exclusion criteria were an age less than 14 and more than 45, any co-morbid conditions other than IBD, cessation of pregnancy due to causes other than IBD. All full-text articles were examined and read thoroughly to reach the accuracy of each article included. What lies in the exclusion criteria is beyond the scope of this literature review and is encouraged to be searched for in the future.

## Review

The human body generally reacts to any stimulus that can alter or disturb the natural environment and this can involve various organs and organ systems. The GI tract is exposed to many insults daily. The wear and tear within the human gut can lead to devastating consequences. The events disturbing the natural habitat of the gut can range from infection to inflammation, and to trauma.

Inflammation can take a chronic course, auto-immunity being a property of such advancing diseases. Such ailments not only involve the primary organ but can also lead to systemic manifestations. A condition highlighted in this article is that of IBD which is a spectrum of chronic idiopathic inflammatory conditions with episodes of relapsing and remitting intestinal inflammation and extra-intestinal manifestations affecting several different regions [1]. Ulcerative Colitis (UC) involves the colonic and rectal mucosa and submucosa while Crohn’s Disease (CD) is characterized by transmural inflammation involving any region but sparing the rectum [1]. Hallmark GI symptoms of IBD include diarrhea, constipation, bloody stools, increased bowel movements, abdominal cramping, nausea, and vomiting. In addition to GI symptoms, fever, weight loss, arthralgias, and malaise are other frequent systemic symptoms seen in IBD. Fistulizing diseases, as well as fat and vitamin malabsorption, are long-standing complications. IBD is usually diagnosed in patients of reproductive age and hence can either present as flare-up or remit during or after conception [2]. Women may avoid a pregnancy due to several reasons like genetic susceptibility, fetal complications, use of various drugs and their interaction to the developing embryo. It is difficult for most patients to stay in complete remission during pregnancy. A shared decision making is needed to progress through the span of conception, with a Gastroenterologist and an Obstetrician to avoid possible complications.

In an article by De Lima et al, it is stated that preconception counseling can help limit the negative impact of pregnancy-related issues and fertility in IBD [3]. This disease needs special attention during pregnancy. The fact is further elaborated by Gawron et al that educating a woman with IBD during her fertile years or during pregnancy includes a multitude of topics like detailed information about her medications, alteration of the existing medications to avoid significant teratogenicity, introduction of nutritional supplements (folic acid, iron, etc.), and emphasizing on the need to keep the disease in sustained remission using appropriate drugs, are a few to highlight on every visit [4].

Genetic course

The genetic course of any disease leads to an uncertainty of whether the disease would progress to the offspring, which can be a contributing factor to avoid pregnancy. IBD is one such ailment among many to fall into this category. The first description of IBD following the genetic route was in the early 20th century when genome-wide association studies found around 200 IBD risk loci [5]. Gordon et al indicate that there is an exceeding 30% chance of two affected parents to give birth to off-springs affected with IBD. Moreover, the most significant risk factor contributing to the disease is the presence of one or more first-degree relatives affected by the disease. Owing to this fact, it is not impossible for the disease to be transmitted within generations [6].

Impact of IBD on Pregnancy

Many physiological and pathological conditions can wax and wane the symptoms of disorders like IBD. One such physiological condition is that of pregnancy. As pregnancy is a high demand and supply state, the mother, as well as the infant, remain at a higher chance of complications. The adverse outcomes related to IBD can be a flare-up of the symptoms, leading to intense diarrhea associated with abdominal pain and bloody stools. This can also lead to malabsorption and anemia. It is of foremost importance that the disease is kept in strict remission and disease activity is kept at a low level. The impact of the disease can be devastating for the mother as well as the developing fetus [2]. IBD has a propensity to be diagnosed more commonly in women than men [7], with about 25% of women conceiving post-diagnosis, frequently in the early years of life that are also peak reproductive years. The disease activity has been identified as a plausible risk factor for complications during pregnancy [8]. Several population-based studies have shown an infertility rate of about 5-14% for CD, as compared to a less impact on fertility in case of UC. This, however, is related to patients who have not undergone IBD related surgeries [9,10].

Impact of IBD on the Developing Fetus

As stated earlier, many known chronic illnesses can have active bouts during pregnancy. Moreover, about 30-40% of women have active IBD during conception with an intense flare-up and adverse outcomes like spontaneous abortions, pre-term and low birth weight (LBW) (<2500 g regardless of gestational age), ischemic placental disease, stillbirth, and caesarian delivery. There is evidence that the disease activity is more detrimental to gestation and consequently to the fetus than the medical therapy itself. Hence, as the first recommendation, the patient should be in disease remission at conception, favoring a pregnancy with lower risks of gestational adversities. Most of the conditions are associated with the maternal milieu and depressed nutrient delivery to the growing fetus [11]. Some studies have demonstrated that rates of fetal loss and preterm birth are higher in patients who conceive while their disease is active. In patients whose IBD was active during pregnancy, higher rates of preterm birth and LBW were noted [12]. A life fed within life can be a challenging situation during disease activity with active symptoms leading to the aforementioned complications. A comprehensive meta-analysis of 12 studies including 3,907 IBD women compared to 320,531 controls showed an increase in preterm birth, LBW, congenital abnormalities, and C-section rates [13]. Likewise, fetal health, both pre- and intra-partum relies on the type, intensity, and extent of the disease, as well as for the medications used during pregnancy [11]. Nutrient supply to the fetus is dependent upon the well-being of the bowel. Any acute or chronic insult can lead to conditions like malabsorption of the vital components for the developing baby, due to the fact of maintaining an environment of supply and demand of nutrients from the mother to the fetus. Nonetheless, drugs and their impact on the growing fetus can also be quite challenging. Although the number of prospective clinical studies focused on the impact of IBD medications during pregnancy is increasing, there is still a marked deficit in our understanding of how active disease elicits immediate pregnancy risks and how maternal disease throughout gestation may impact fetal immune development and lifelong IBD susceptibility [1]. In a Swedish cohort, women with IBD had high rates of postpartum admissions for GI indications leading to stillbirths. A discrepancy lies with the impact of pregnancy in IBD. It is thought to either be due to delayed investigations or stopped treatment either during pregnancy or lactation [8]. A few studies illustrated the outcomes of IBD during pregnancy, one such study known as pregnancy loss cohort, which states no difference in the number of miscarriages in patients with IBD. Another, congenital abnormality cohort suggests that there is an increased risk of major congenital abnormality associated with CD [14]. Another study by Stephansson et al describes an association of IBD with low APGAR score suggesting the infants born to mother’s with IBD can have greater distress and ultimately lower APGAR at least immediately after birth [15]. Several population‐based case-control studies have reported no increase in stillbirth, neonatal death or spontaneous abortion [16]. Emphasized above, patients with IBD should be maintained using appropriate medication. Most of them have a low risk to the fetus, except for a few like Methotrexate and Thalidomide [11].

Maternal Morbidity and the need for Surgical Intervention

Maternal well-being is needed for the survival of the developing fetus. The disease course of IBD can be a limitation in this scenario. The most important factor leading to adversities is the possibility of conceiving. There is a concern of IBD and its relation to fertility as it remains a major topic of concern in a young female of newly diagnosed or established IBD. On the contrary, UC does not have diminished fertility rate on the whole. Moreover, patients with UC who have undergone bowel surgery may present with complications with conceiving [17,18]. Of note here is the impact of this chronic disease on the ovarian reserve. Females over the age of 30 with CD, were at a higher risk of reduced ovarian reserves. Most of the individuals had CD involving the colon at most [19]. Molnar et al conducted a case-control study to compare the outcomes in women who conceived before and after the diagnosis of IBD. In this study, it was evaluated that the risk of preterm birth and LBW were more common in women who were diagnosed with IBD following pregnancy [20]. CD remains one of the reasons for preterm birth. Leading to this fact, a link can be found with CD and LBW [21]. There is also a four-fold increased risk of Venous Thromboembolism (VTE) reported in women with UC. Conservative medical management while monitoring maternal nutrition and prenatal care with prophylactic heparin can prevent devastating consequences leading to VTE. Likewise, the risk of antepartum hemorrhage is shown to double in women with CD [2,22]. One study elaborated the outcomes of IBD in remission and pregnancy. A majority (~80%) female patients remained in remission in the intrapartum and postpartum period. About 23 patients with IBD conceived when their disease was active, of which, 66% continued to have disease activity or worsening of their symptoms. In up to 45% of patients with UC who conceived while their disease was active, the colitis worsened during pregnancy; in 24%, the colitis continued to be active but stable, and in the remainder of patients, the disease went back into remission [23]. In addition to the symptoms illustrated above, several other factors may contribute to a reduced rate of conception in patients with active IBD. These can present as psychological complications of chronic illness and can include dyspareunia, low libido, and depression [2]. A study conducted in the United States affirms that CD itself is a risk factor for adverse events requiring surgical interventions in pregnant women with CD [24]. Hence, this remains a negative impact on fertility, widely ranging with the type or extent of the surgery [25].

Inflammatory Bowel Disease and Caesarian-Sections

A variety of obstetric complications can develop during an otherwise normal pregnancy which may lead to attempts on aggressive measures. This can also prevail in women with IBD. The disease and its related complications can narrow down the choice between vaginal deliveries versus cesarean sections. A study conducted by Cornish et al demonstrated that women with UC have a cesarean section rate compared with the general population unlike women with CD, who are more prone to undergo C-sections [13]. Bradford et al illustrate that C-sections remains more prevalent among women with IBD, most likely due to peri-anal disease, in addition to the other obstetric complications [26]. According to the European Crohn's and Colitis Organization, C-section remains a mandate in women who develop the active perianal disease during pregnancy, owing to the increased risk of complications to both the mother and the child. However, women who are either in remission or have not developed perianal complications from CD can undergo vaginal deliveries and episiotomies, as they are unlikely to develop perianal disease postnatally [27].

IBD Drugs and its Impact on Pregnancy

As disease activity remains the most important factor of outcomes during conception. The mode by which it can remain in sustained remission is by the apt use of medications. A multitude of drugs is available in the market for its use in patients with IBD. Keeping in view the impact of these drugs on the developing fetus, the Food and Drug Administration (FDA) has formulated a set of five distinguishing categories, each drug comprising its effect on the potential for causing different birth defects if consumed during pregnancy [28]. Many of these medications used in the control of IBD fall into this field. As drugs have different modes of actions, interfering either in the enzyme pathway or acting directly on TNF- alpha, modulating the maternal as well as the fetal immune system, additional supplements are introduced to mothers on these medications. A few to mention are avid use of Iron, folic acid, etc to minimize the influence of these drugs on fetal development. Additionally, a cautious prescription is needed to avoid any future problems with the growing fetus.

*Sulfasalazine:* Sulfasalazine is an FDA Category B drug used in pregnancy. This drug is known to function in a number of ways, one such mechanism is inhibition of the compounds on the cyclooxygenase and lipoxygenase pathway. It also scavenges free-radicals owing to its anti-inflammatory action on the gut. Interference with the enzymatic action of folate can lead to fetal complications like neural tube defects [29]. The adverse effects due to this drug can be minimized using supplemental folate in the quantity of 5 mg as compared to 0.4mg in the general population. The drug and its metabolites can freely pass through the placenta leading to the fetal complications. Moreover, the sulfa component in the drug can displace bilirubin from albumin leading to kernicterus in the newborn. However, this drug can be safely used in pregnancy provided safety measures are considered. Very minimal amounts of the drug are excreted in breast-milk of the lactating mother, considering it safe during breastfeeding [30]. Mesalazine has been classified as FDA category B and C for pregnancy. Mesalazine is broken down to its active metabolite N-acetyl mesalazine by the liver; this then crosses freely through the placenta. Due to the rapid clearance of this chemical by the kidneys, it does not cause significant harm to the fetus. Low levels can be found in breast milk, deeming it to be safely prescribed during lactation [30].

*Metronidazole: *Metronidazole is FDA pregnancy category B antibiotic. It is one of the most commonly prescribed medications in IBD, inhibiting nucleic acid synthesis by disruption of microbial cell deoxyribonucleic acid (DNA) [31]. The drug has a propensity to cause cleft lip in the initial days of pregnancy. If used in dosage over 2gm, it is excreted in large quantities in the breast milk. It is highly advised for women on single high doses to avoid breastfeeding for at least 12-24 hrs. However, due to this reason, it is advisable to avoid the drug during breastfeeding [32].

*Quinolones: * The drug is FDA category C drugs for pregnancy, inhibiting bacterial type II topoisomerases, gyrases, and topoisomerase IV enzymes [33]. It has a propensity to accumulate in the joints and bones. Furthermore, it is also excreted in breast-milk. Due to this, it is advisable to avoid the drug during pregnancy and an alternative should be prescribed [30]. Studies on humans have not shown an increase in miscarriages or congenital abnormalities. Nonetheless, many animal studies suggest musculoskeletal abnormalities. The drug has an affinity for cartilages and bones causing arthropathies in children. Despite studies suggesting a negligible risk of adverse outcomes, it is advised to avoid the drug in the first trimester [11].

*6 Mercaptopurine/Azathioprine: *Immunomodulators like 6 Mercaptopurine/Azathioprine (6MP/AZA) are classified as FDA category D drugs. By inhibiting purine synthesis, nucleotide interconversions, DNA and RNA synthesis, and chromosomal replication [34], these drugs are known to cause maternal as well as fetal complications, as they are detected in the fetal blood, with as high as 5% of the maternal drug level. 6MP can cause serious bone marrow and liver toxicity and can also lead to pre-term delivery. On the other hand, since the drug modulates the immune system, it can also lead to immune-compromise in fetuses of mothers on this therapy during pregnancy. Likewise, the baby can be born with an LBW; can be small for gestational age with various congenital malformations. Moreover, the drug is teratogenic in nature [30,35].

*Methotrexate: *Methotrexate acts by Inhibiting enzymes in the metabolic pathway of folic acid interfering in purine and pyrimidine synthesis [26], making it devastating for both the mother and the fetus. The drug is categorized as FDA class X. It is advised to avoid pregnancy while on the drug. Exposure to methotrexate can lead to several complications, including miscarriage, growth retardation, fetal loss and congenital malformations, including craniofacial anomalies, limb defects, and central nervous system (CNS) abnormalities. The active metabolite has a half-life of around six weeks to reach a steady-state or for a complete wash-out. However, couples are advised to use a barrier method of contraception in order to avoid pregnancy. Termination of either pregnancy or the drug supplementing high dose folate is required if conception does occur. The drug has the capability of passing through the placenta as well as breast milk, hence, it is avoided during lactation also [11,30].​​​​​​​

*Cyclosporine/Tacrolimus: *Immune Suppressants like Cyclosporine/Tacrolimus are medications widely used in the transplant recipients are FDA category C for pregnancy and function by T-cell activation inhibition, decrease in the expression of nuclear factor of activated T-cell (NFAT) -regulated cytokines, including interleukin-2 (IL-2), interferon-gamma (IFN-γ), and tumor necrosis factor-alpha (TNF-α) [36]. These medications lead to the suppression in the immune system by acting on various pathways and are mostly used during drug-resistant IBD. Cyclosporine A has a rapid onset of action, reducing the disease activity much earlier than the rest of the drugs. A meta-analysis including 15 studies and 410 patients receiving cyclosporine during pregnancy found an odds ratio of 3.83 for malformations, which was, however, not statistically significant. The overall prevalence of major malformations in infants whose mothers were treated with cyclosporine during pregnancy was 4.1% and did not differ from that in the general population. Although the prevalence rate for preterm birth was 53.6% (from a total of 10 studies, including 379 patients), the OR did not reach statistical significance. Likewise, mostly used in UC, tacrolimus exerts immunosuppressive actions, by inhibiting T-cell activation and proliferation by targeting the calcineurin pathway [37]. As it does not require biliary or mucosal integrity for absorption, it can be used in patients with small bowel involvement. Most importantly, the drugs are safe to be used during lactation as they minimally cross the breast milk [11,30].​​​​​​​

*Steroids:* The most widely used drug in a majority of ailments, are FDA category C for pregnancy. They are known to act through three main mechanisms; direct effects on gene expression by the binding of glucocorticoid receptors to glucocorticoid-responsive elements. Indirect effects on gene expression are through the interactions of glucocorticoid receptors with other transcription factors. Glucocorticoid receptor-mediates effects on second-messenger cascades [38]. Most clinicians use this drug at times an active flare-up in IBD. Steroid use can lead to long term complications. Individuals prescribed with the drug have no known fertility risks. However, the impact on maternal and fetal morbidity cannot be excluded. A few studies suggest an increased incidence of oro-facial malformations in fetuses of mothers taking steroids especially in the first trimester of pregnancy. A few reported cases show neonatal adrenal suppression and premature rupture of placental membranes due to the use of corticosteroids late in pregnancy by women with IBD. It remains important to consider the risks and benefits when prescribing steroids to the mother. Most of the maternal risks are similar to a non-pregnant status which includes complications such as hypertension, diabetes, and preeclampsia seen to be increased during pregnancy. This drug also has unfavorable pregnancy outcomes. Glucocorticoid use during lactation is generally considered to be safe [2,11,30].

*Biologics:* TNF alpha Inhibitors/ IL-23 Antibody/Anti-integrins suppress the physiological response of the body to tumor necrosis factor (TNF) and inhibit the pro-inflammatory agents involved in chronic inflammation to reduce the progression of the disease [39]. These drugs have been satisfactorily used in various other conditions. This group of medications falls in FDA category B drugs for use during pregnancy. The anti-integrin drug Natalizumab is category C [30]. Biologics are a new entity of medications used to keep symptoms of IBD in sustained remission. Few of them included are infliximab, adalimumab, certolizumab pegol, and golimumab. A number of studies suggest no clear association between treatment with biological agents for IBD in pregnancy and adverse pregnancy outcomes, even if administered during the third trimester. The Pregnancy in Inflammatory Bowel Disease and Neonatal Outcomes (PIANO) registry intends to determine the adverse outcomes among pregnant women with IBD and their offsprings on AZA, 6MP and anti-TNF biologics. The registered women are evaluated during every trimester, on delivery and the initial four years of the baby’s life. A record of newborn complications, maternal disease activity and complications, medications and developmental milestones in the offspring are noted. The impact of these drugs is compared with a control group of IBD patients without immune-suppressive medication or anti-TNF treatment. Currently, 1564 patients have been enrolled in this study making it one of the largest prospective registries conducted in 31 IBD centers in the United States looking for maternal and childhood outcomes in IBD [2,11,40]. A study conducted by Vermiere S et al showed that there is a low risk of pregnancy complications with anti-TNF therapy, especially when used in the first two trimesters and during preconception [41]. Mahadevan et al also describes the safety profile of anti-TNF agents during pregnancy and does not cause adverse events [42]. The serum level can be detected for up to six months in the neonates. Live attenuated vaccines like rota, oral polio, and BCG are not indicated postponing them for six months or more in children exposed to TNF agents during gestation [43]. Owing to the large molecular weight if these drugs, negligible levels are found in the breast milk and are therefore considered safe during lactation [1]. A study conducted in the Czech Republic illustrated an exposure to anti-TNF-α antibodies seemed to be safe for the growth and psychomotor development of children [44]. A study done by Heller et al looked at 495 women exposed to a TNF inhibitor early in pregnancy (almost one-third of the women were taking infliximab) did not find an increased risk for miscarriage [45]. An IL-12/23 humanized anti-body Uztekinumab bears insufficient data to be prescribed during pregnancy and lactation. It is indicated to discontinue the drug about 15 weeks prior to gestation. To date, there is no evidence of Uztekinumab passage in breast milk. Due to insufficient data on Vedolizumab another humanized IgG1 antibody, it is avoided during pregnancy and lactation [46].

## Conclusions

In summary, IBD is a disease diagnosed mostly in women of child-bearing age and can lead to unnecessary adversities. The disease should be kept in sustained remission to limit the morbidity and mortality in the mother and the fetus. It should be emphasized to have a regular pre-conception and intrapartum obstetric followups. The nutrition of the mother should also be kept under consideration with the introduction of supplemental vitamins and minerals. In addition to this, the medications used to keep the activity of the disease low should be regulated according to their side effects.
